# Clinical Characteristics and Outcome of Readmitted Adult Patients With Acute COVID-19 Infection Within 30 Days of Their Hospital Discharge

**DOI:** 10.1155/cjid/8843908

**Published:** 2025-05-27

**Authors:** Nawaf Abdulaziz Alobaid, Ali Abdulrahman Alsalamah, Mohmmed Ibrahim Mugren, Abdulaziz Mohammed Alhwairini, Mohammed Ali Alzahrani, Nawaf M. Alzahrani, Omar Baharoon, Jinan Shamou, Eiman Alsafi, Salim Baharoon

**Affiliations:** ^1^College of Medicine, King Saud Bin Abdulaziz University for Health Sciences, Riyadh, Saudi Arabia; ^2^King Abdullah International Medical Research Center, Riyadh, Saudi Arabia; ^3^Ministry of National Guard-Health Affairs, Riyadh, Saudi Arabia; ^4^Department of Medicine, King Abdulaziz Medical City, Riyadh, Saudi Arabia; ^5^College of Medicine, Dar Al-Uloom University, Riyadh, Saudi Arabia; ^6^King Saud Bin Abdulaziz University for Health Sciences, Riyadh, Saudi Arabia; ^7^Al Yamamah Hospital, Riyadh Second Health Cluster, Ministry of Health, Riyadh, Saudi Arabia

**Keywords:** COVID-19, readmission, readmission rate, risk factors

## Abstract

**Introduction:** Readmission to the hospital after an acute COVID-19 infection varies in the literature in terms of rate, causes, and outcomes. The 30-day readmission rate ranges from 4% to as high as 11.3%. The causes of readmission after a COVID-19 admission are diverse and include persistent respiratory symptoms, hypoxia, secondary bacterial infection, and thromboembolic disease. This study aims to describe the causes of hospital readmission within 30 days of discharge following an acute COVID-19 infection.

**Methods:** This retrospective cohort study was conducted at a tertiary care center in Riyadh, Saudi Arabia, between March 2020 and February 2022 and included all adult patients who were readmitted to the hospital within 30 days after a primary hospital admission due to COVID-19 infection.

**Results:** A total of 3517 patients were hospitalized with acute COVID-19 infection during the study period, and 200 patients were rehospitalized within 30 days postdischarge, resulting in a readmission rate of 5.7%. The mean age of the readmitted patients was 66.35 ± 19.5 years, and 105 (52.5%) were male. Hypertension and diabetes mellitus were the most common comorbidities. Chronic respiratory disease was present in 44 patients (22%) prior to their acute COVID-19 infection. The mean time to readmission was 7.86 ± 5.8 days. Persistent COVID-19 pneumonia was the most common cause of readmission, diagnosed in 105 patients (52.5%), followed by renal impairment in 29 patients (14.5%). Urinary tract infections were the leading infectious cause of readmission, occurring in 23 patients (11.5%), while secondary bacterial pneumonia was rare. Shortness of breath and cough were the most common symptoms at the second presentation. Respiratory therapeutic interventions were required for 120 patients (60%), and 45 patients required intensive care unit (ICU) admission. Compared to the index admission, a higher proportion of patients required ICU admission and mechanical ventilation. After the index admission, most patients were still symptomatic at discharge (moderate to critical National Early Warning Scores (NEWS)).

**Conclusion:** The readmission rate after acute COVID-19 infection was 5.7%, aligning with rates reported internationally. The most frequent causes of readmission were persistent COVID-19 pneumonia, renal impairment, and urinary tract infections, while secondary bacterial pneumonia at readmission was rare. Readmission was associated with increased rates of ICU admission and the need for mechanical ventilation. The use of NEWS at discharge may serve as a useful criterion for determining readiness for discharge. Future follow-up of this cohort of patients will determine chronic long-term respiratory complications.

## 1. Introduction

The COVID-19 pandemic is one of the most significant health crises of the 21st century. The virus was first identified in Wuhan, China, in December 2019, and the World Health Organization (WHO) officially declared it a pandemic on March 11, 2020 [1]. Since then, countries worldwide have faced substantial health and economic consequences.

Severe acute respiratory syndrome coronavirus 2 (SARS-CoV-2) predominantly affects the respiratory tract, with acute pneumonia and acute respiratory distress syndrome (ARDS) being among the most severe complications [2]. Both acute and chronic sequelae of COVID-19 infection have been reported, including an increased risk of thromboembolic disease, bowel ischemia, severe ileus, acute ischemic stroke, acute liver and kidney injury, and arrhythmias, among many others [3–8].

The rate and causes of hospital readmission following an acute COVID-19 infection vary depending on the definition of readmission used, geographical location, patient characteristics, and the timing of the study relative to the pandemic's onset. For example, in China, the 30-day postdischarge readmission rate was reported to be less than 8.8% [9], while one center in northern Saudi Arabia reported a rate of 5.9% [10]. Similarly, studies in the USA have shown a readmission rate of around 6.4% [11].

Notably, a higher readmission rate of 11.3% has been observed among individuals aged 80 years and older [1]. Identified risk factors for readmission include preexisting respiratory disease, intensive care unit (ICU) admission, advanced age, elevated creatinine levels at discharge, cancer, high body mass index (BMI), and chronic obstructive pulmonary disease (COPD), among others [2, 10, 12–16].

In the current study, we investigated the rate and causes of hospital readmission within 30 days following an initial COVID-19 admission. Specifically, we analyzed the most common causes for readmission and explored whether comorbidities were associated with a more severe disease course requiring a second hospitalization. Additionally, we assessed the clinical presentation, hospital course, length of stay, and mortality during the second admission.

## 2. Methods

This retrospective chart review included all adult readmissions at King Abdulaziz Medical City (KAMC) within 30 days of a primary admission for COVID-19 infection between March 2020 and February 2022. KAMC, located in Riyadh, is a major tertiary care center with a total bed capacity exceeding 2000 and played a critical role in managing COVID-19 cases in Saudi Arabia during the pandemic.

The inclusion criteria were patients aged 18 years or older, with a primary admission due to acute respiratory COVID-19 infection, confirmed by at least one positive COVID-19 PCR result from a respiratory sample, and subsequent readmission within 30 days of discharge.

Patients under the age of 18 years, as well as pregnant females with a confirmed COVID-19 test who were scheduled for follow-up appointments, were excluded from the study. Several challenges were encountered with specific files. For instance, some patients chose to discharge themselves against medical advice early during readmission, which limited the collection of key variables. Additionally, the National Early Warning Scores (NEWS) depends on specific parameters that are critical for the study's accuracy and completeness; therefore, patients with missing or incomplete parameters were excluded. Moreover, certain values were unavailable in physician notes. However, this issue was mitigated by cross-referencing nursing notes to ensure data completeness and reliability. Despite these challenges, careful efforts were made to minimize data gaps and maintain the integrity of the study.

Data were manually extracted from electronic patient medical records using the BestCare system by a team of six trained research assistants under the supervision of the principal investigator. To ensure consistency and accuracy, a standardized electronic data collection form was developed. The extracted variables for primary admissions included patient demographics (age, sex, and BMI), comorbidities (e.g., hypertension, diabetes mellitus, cardiovascular disease, chronic lung disease, chronic kidney disease, malignancy, and immunosuppression), symptoms at presentation (e.g., cough, fever, dyspnea, and fatigue), and vital signs upon admission (respiratory rate, oxygen saturation, temperature, systolic blood pressure, heart rate, and consciousness level), Laboratory investigations encompassed urea and creatinine levels. Additionally, data on antibiotics or antivirals administered for COVID-19 and radiological findings from chest X-rays (presence or absence of infiltrates, unilateral or bilateral infiltrate distribution, and pleural effusion) were collected.

The severity of COVID-19 infection at both admission and discharge was assessed using the National Early Warning Score 2 (NEWS2). This standardized tool, developed by the Royal College of Physicians in 2012, is designed to detect and respond to clinical deterioration in adult patients. NEWS2 assigns scores to routinely measure physiological parameters, including respiratory rate, oxygen saturation, temperature, systolic blood pressure, heart rate, and consciousness level. Higher scores indicate greater deviation from normal ranges. Severity was categorized as low (score 0–2), moderate (score 3–5), high (score 6-7), or critical (≥ 8).

Kidney function was categorized as “normal,” “abnormal,” or “known abnormal” based on serum creatinine levels. Abnormal kidney function was defined by serum creatinine levels exceeding the upper limit of normal according to laboratory reference ranges (> 106 μmol/L in males and > 97 μmol/L in females). Known abnormal kidney function included patients with a prior diagnosis of chronic kidney disease or those who were discharged from their initial admission with abnormal creatinine levels that persisted until their second admission.

For hospital readmissions, data collection included the primary causes of readmission (categorized as respiratory, cardiovascular, infectious, renal, or other), presenting symptoms, patient disposition, radiological findings, urea and creatinine levels, and respiratory support requirements. Respiratory support was classified into five levels: no oxygen, low-flow oxygen, high-flow nasal cannula, noninvasive ventilation, and invasive mechanical ventilation. Additional data included ICU admission status, length of ICU and hospital stay, and outcomes (discharged home, mortality, or transfer). The NEWS2 was also recalculated at readmission to assess the severity of the patient's condition.

To maintain data accuracy, a random sample of 10% of patient charts underwent independent review by two research assistants. Inter-rater reliability was measured using Cohen's kappa coefficient, with values exceeding 0.8 for all key variables.

Potential limitations associated with manual data extraction from the BestCare system include human error, variability in documentation quality, and incomplete medical records. To minimize these risks, data extractors received rigorous training and used standardized data collection forms, and discrepancies were resolved through regular consensus meetings.

The study was approved by the hospital's ethics committee and the King Abdullah International Medical Research Center (KAIMRC) under approval number IRB/2086/22. Informed consent was not required due to the retrospective nature of the study.

### 2.1. Statistical Analysis

The data were initially entered into Microsoft Excel and subsequently exported to the Statistical Package for the Social Sciences (SPSS) version 25 for analysis. Categorical variables were reported as frequencies and percentages, while continuous variables were presented as mean ± standard deviation or median (interquartile range) as appropriate. Relationships between variables were analyzed using the chi-square test or Fisher's exact test, depending on suitability. For continuous data, two independent sample *t*-tests (unpaired *t*-tests) and the nonparametric Mann–Whitney *U* test (Wilcoxon rank-sum test) were used as appropriate. A *p*-value of < 0.05 was considered statistically significant.

## 3. Results

Between March 2020 and February 2022, a total of 3517 patients diagnosed with acute COVID-19 infection were hospitalized at KAMC. Of these patients, 200 were rehospitalized within 30 days following their initial discharge, resulting in a readmission rate of 5.7%. The mean time to readmission was 7.86 ± 5.8 days. The mean age of patients readmitted was 66.35 ± 19.5 years, and the male-to-female ratio was 1.1:1 (Table 1).

The most frequently observed comorbidities among readmitted patients were hypertension (137 patients, 68.5%), diabetes mellitus (131 patients, 65.5%), and chronic cardiovascular diseases (74 patients, 37%). Specific cardiovascular diseases included heart failure (56 patients, 28%) and atrial fibrillation (18 patients, 9%). Chronic kidney disease was documented in 55 patients (27.5%), with 25 of these patients (45%) requiring regular hemodialysis. Chronic respiratory disease was identified in 44 patients (22%), of whom 34 (17%) had bronchial asthma and 10 (5%) had COPD. A history of thromboembolic disease was noted in 12 patients (6%) (Table 1).

Persistent COVID-19 pneumonia (defined as ongoing COVID-19 compatible symptoms with hypoxia not attributable to other conditions) was the most common reason for readmission, present in 105 patients (52.5%). Other documented reasons included urinary tract infections (UTIs) (23 patients, 11.5%), bacterial pneumonia (1 patient, 0.5%), and acute kidney function deterioration (15 patients, 7.5%). Additionally, 14 patients with chronic kidney disease experienced worsening renal function compared to their status at discharge.

Other reasons for readmission included pulmonary edema or fluid overload (13 patients, 6.5%), new ischemic cardiac events—two cases of ST-elevation myocardial infarction (STEMI) and three cases of non-STEMI—one cardiac arrest event, new-onset atrial fibrillation (18 patients, 9%), thromboembolic events (7 patients; 5 pulmonary embolism and 2 deep vein thrombosis (DVT)), and stroke (3 patients, 1.5%). Acute cholecystitis (6 patients, 3%), acute liver dysfunction (2 patients, 1%), and acute pancreatitis (2 patients, 1%) were also noted. Six patients were admitted electively for hemodialysis, and other elective admissions included labor, heart transplant evaluation, fetal monitoring, and malignancy assessment.

ICU admission was required during readmission in 45 patients (22.5%), compared to 24 during initial admission. Intubation and mechanical ventilation were required in 19 patients (9.5%), while noninvasive respiratory support (BiPAP or high-flow nasal cannula) was needed in 88 patients (44%). COVID-19 testing was repeated in 123 patients (61.5%), with 120 (97.5%) maintaining a positive test result.

Shortness of breath and cough were the most frequent symptoms upon readmission (Table 1). Compared to initial admissions, significantly fewer patients reported cough (91 patients, 45.5% vs. 59 patients, 29.5%; *p* < 0.001) and fever (44% vs. 23.5%; *p*=0.001). There were no significant differences regarding shortness of breath, chest pain, or gastrointestinal symptoms between the two admission episodes (Table 2). Oxygen desaturation at admission increased from 22% during initial admissions to 36.5% during readmission. Respiratory interventions in the emergency department increased significantly from 90 patients (45%) at initial admission to 120 patients (60%) at readmission. Specifically, 64 patients (32%) required high-flow nasal cannula at readmission compared to 60 patients (30%) at initial presentation.

According to the NEWS2 during the initial admission, 123 patients (61.5%) were categorized as critical, with 23 patients classified as having high scores. At the time of discharge from the initial admission, only 39 patients (19.5%) remained in the critical category. Upon readmission, 128 patients were classified as critical, and 21 had high NEWS2 (Table 3).

The median (IQR) ICU stay during readmission was 8 days (3.5–23.5), compared to 4 days (1–12) during the initial admission. The median hospital length of stay also increased from 5 days (3–10) at the index admission to 8 days (4–14) during readmission. Mortality during the second hospitalization was documented in 25 patients (12.5%), with respiratory failure (with or without sepsis) identified as the predominant cause in 17 patients (68%).

## 4. Discussion

Hospital readmission following discharge is a critical indicator for evaluating healthcare delivery quality and efficiency. This observational study found a readmission rate of 5.7%, consistent with international findings reported in similar COVID-19 cohorts. Most readmissions occurred relatively early, with a mean interval of approximately 7.5 days postdischarge, aligning closely with prior studies that reported readmissions typically within 5–7 days [2, 18, 19]. Higher readmission rates have been documented among specific subgroups, notably individuals aged over 80 years [1, 16, 19, 20]. The observed similarity between our findings and those from other regions of Saudi Arabia suggests a possible national trend [10].

Persistent COVID-19 pneumonia was the primary cause of readmission in over half of the patients (52.5%), exceeding figures reported in other studies (approximately 30%) [21], although comparable to higher estimates (68.8%) observed elsewhere [2]. Interestingly, only 22% of our cohort had preexisting respiratory conditions, suggesting ongoing COVID-19-related lung involvement rather than exacerbation of chronic lung disease as the predominant driver of readmissions.

Acute kidney injury and UTIs represented other leading causes of readmission. The occurrence of acute renal impairment upon readmission (14.5%) was within the wide range (0.5%–46%) documented globally, with geographical variability likely reflecting differences in healthcare practices, patient demographics, and possibly circulating SARS-CoV-2 variants [5, 22–24]. Although UTIs are a common admission reason in Saudi Arabia independent of COVID-19, recent studies suggest a potential pathophysiological link through ACE-2 receptor expression in the urinary tract, thus possibly connecting UTI occurrence directly with SARS-CoV-2 infection [25–27].

DVT occurred at a substantially lower rate (0.1%) compared to previously reported rates (up to 12%) [28]. This discrepancy may be attributable to population-specific characteristics, adherence to thromboprophylaxis protocols, or variations in reporting standards across studies.

Our findings also indicated a notable increase in ICU admissions and invasive respiratory support requirements upon readmission. This may reflect the progression of COVID-19-related respiratory disease or an increased risk of associated complications, such as secondary pneumonia or heart failure. Reported ICU admission rates and invasive ventilation requirements in the literature vary significantly (7%–60%), reflecting the diverse clinical severity, patient demographics, and follow-up durations across different studies [21, 29, 30].

The observed mortality rate during readmissions was substantial (12.5%), with respiratory failure and sepsis being the most common underlying causes. These findings closely match other international reports, documenting postdischarge mortality ranging between 7.2% and 17.9% within 30 days after initial hospitalization [20]. The 1-year postdischarge all-cause mortality rate is estimated at 7.87%, with most deaths occurring within the first 30 days after discharge [31].

The relatively high mortality observed among our readmitted patients likely stems from multiple factors: underlying comorbidities, the severity of initial infection, and potentially premature discharges related to healthcare system constraints during the pandemic. The NEWS2 has been used during the COVID-19 pandemic as a sensitive tool for severity assessment and to identify patients at risk of clinical deterioration [17, 32, 33]. Notably, a large proportion of patients discharged (83%) had moderate to critical NEWS2, raising concerns about the criteria used for discharge decision-making. Literature has identified low NEWS2 at discharge as protective against readmissions [34]; thus, higher scores observed in our patients warrant further investigation into discharge planning practices at our facility.

While NEWS2 scoring systems are not currently standardized for discharge decision-making regarding COVID-19, this study highlights their potential utility. Furthermore, exploring additional biomarkers such as D-dimer, lactate dehydrogenase, and neutrophil-to-lymphocyte ratio at discharge could further enhance risk stratification and potentially reduce readmission rates through more targeted postdischarge follow-up or delayed discharge for high-risk patients [35].

The generalizability of our findings warrants careful consideration. Our study was conducted in a single tertiary healthcare center in central Saudi Arabia, potentially limiting applicability to other healthcare settings or geographical regions. Differences in population demographics, healthcare infrastructure, local clinical practices, and SARS-CoV-2 variant distribution can significantly influence the generalizability of these results. However, our findings appear broadly consistent with national and international data, enhancing their potential relevance beyond the immediate setting. Future studies in diverse healthcare environments, incorporating variant surveillance and longitudinal follow-up, will be crucial for validating and refining our observations.

### 4.1. Limitations

Several important limitations are acknowledged in this study. Firstly, the single-center design may limit wider applicability. Secondly, while overall readmission rates were calculated, temporal variations during the pandemic were not assessed, potentially masking dynamic changes related to evolving clinical practices, circulating viral variants, or vaccination coverage. Additionally, the lack of identification of specific SARS-CoV-2 variants circulating during the study period restricts a more nuanced understanding of disease severity and readmission patterns. These limitations emphasize the need for future multicenter, prospective studies that evaluate variant-specific impacts on readmissions and systematically assess discharge criteria effectiveness.

## 5. Conclusion

Persistent COVID-19 pneumonia was the most common cause of readmission following hospital discharge, followed by renal impairment and UTIs. Readmission was associated with a higher need for ICU care and increased mortality. Using the NEWS2 scoring system as a discharge tool warrants further exploration as a potential predictor of postdischarge outcomes and to help guide appropriate discharge timing.

## Figures and Tables

**Table 1:** Patients' demographic features and causes of readmission.

Parameter	Frequency *N* = 200	Percentage 100
Age (mean ± SD)	66.35 ± 19.5	
Gender		
Male	105	52.5
Female	95	47.5
BMI (mean ± SD)	28.1 ± 7.5	
Duration between discharge and readmission (days)	7.86 ± 5.8	
Comorbidities		
None	13	6.5
Chronic cardiovascular disease	74	37
Heart failure	56	28
Atrial fibrillation	18	9
Chronic kidney disease	55	27.5
Chronic kidney disease with hemodialysis	25	12.5
Chronic kidney disease without hemodialysis	30	15
Chronic liver disease	8	4
Chronic respiratory disease	44	22
Bronchial asthma	34	17
COPD	10	5
Diabetes mellitus	131	65.5
Hypertension	137	68.5
Hypothyroidism	19	9.5
Malignancy	33	16.5
Previous stroke	25	12.5
Thromboembolic	12	6
Dementia	14	7
COVID-19 test on discharge from index admission		
Positive	42	21
Negative	6	3
Not done	152	76
COVID-19 test on readmission		
Positive	120	60
Negative	3	1.5
Not done	77	38.5
Causes of readmission		
Persistent COVID-19 pneumonia	105	52.5
Bacterial pneumonia	1	0.5
Aspiration pneumonia	1	0.5
Pulmonary edema/volume overload	13	6.5
Pulmonary embolism	5	2.5
Myocardial infarction	5	2.5
STEMI	2	1
Non-STEMI	3	1.5
Acute kidney injury	15	7.5
Acute-on-chronic kidney injury	14	7
Acute pancreatitis	2	1
Acute cholecystitis	6	3
Acute liver injury	2	1
Urinary tract infection	23	11.5
Deep vein thrombosis	2	1
New stroke	3	1.5
Elective hemodialysis session (for patients on chronic dialysis)	6	3
Others^∗^	11	5.5
Symptoms at readmission (multiple options)		
None	24	12
Chest pain	14	7
Confusion	13	6.5
Cough	59	29.5
Fever	47	23.5
Gastrointestinal symptoms	44	22
Headache	2	1
Myalgia	11	5.5
Poor oral intake	23	11.5
Slurred speech	2	1
Shortness of breath	94	47
Vital signs at readmission (mean ± SD)		
Systolic blood pressure	105.6 ± 19.8	
Diastolic blood pressure	60.7 ± 14.5	
Temperature	37.5 ± 0.73	
Heart rate	102.7 ± 21.2	
Respiratory rate	28.1 ± 9.3	
Readmitted patients' NEWS2		
Low	16	8
Moderate	35	17.5
High	21	10.5
Critical	128	64
Hospital course at readmission		
ICU admission	45	22.5
Respiratory support treatment at readmission		
Mechanical ventilation at readmission	19	9.5
BiPAP/high-flow nasal cannula at readmission	88	44
ICU length of stay on readmission, median (IQR)	8 (3.5–23.5)	
Hospital length of stay on readmission, median (IQR)	8 (4–14)	
Causes of mortality at readmission	25	12.5
Respiratory failure	7	3.5
Respiratory failure with sepsis	10	5
Advanced liver disease	4	2
Advanced pancreatic cancer	2	1
Hemorrhagic shock	1	0.5
Unknown (death occurred outside the hospital)	1	0.5

^∗^Others: pancreatic cancer, lung biopsy, labor, recall due to low calcium level, pregnancy-related abdominal pain, PermCath exchange, chemotherapy, heart transplant workup.

**Table 2:** Comparison of clinical presentation between index admission and readmission.

Parameter	Index admission *n* (%)	Readmission *n* (%)	*p*-value
Symptoms			
None	13 (6.5)	24 (12)	0.011
Chest pain	16 (8)	14 (7)	0.201
Confusion	13 (6.5)	13 (6.5)	1
Cough	91 (45.5)	59 (29.5)	**< 0.001**
Fever	88 (44)	47 (23.5)	0.001
Gastrointestinal symptoms	43 (21.5)	44 (22)	0.56
Headache	4 (2)	2 (1)	1
Myalgia	19 (9.5)	11 (5.5)	0.96
Poor oral intake	25 (12.5)	23 (11.5)	0.071
Slurred speech	3 (1.5)	2 (1)	1
Shortness of breath	98 (49)	94 (47)	**0.67** ^**∗∗**^
Vital signs (mean ± SD)			
Systolic blood pressure	109 ± 21	105.6 ± 19.8	0.102
Diastolic blood pressure	61 ± 15	60.7 ± 14.5	0.894
Temperature	37.6 ± 0.8	37.5 ± 0.73	0.079
Heart rate	99 ± 18	102.7 ± 21.2	0.065
Respiratory rate	26 ± 7	28.1 ± 9.3	0.005
O_2_ saturation			
≥ 96%	82 (41)	66 (330)	**< 0.001**
95%–94%	44 (22)	35 (17.5)	
93%–92%	30 (15)	26 (13)	
< 91%	44 (22)	73 (36.5)	
Respiratory therapy			
None	110 (55)	80 (40)	0.568
Oxygen therapy	7 (3.5)	13 (6.5)	0.62
High-flow nasal cannula	60 (30)	64 (32)	0.098
NIPPV/BiPAP	16 (8)	24 (12)	0.029
Mechanical ventilation	7 (3.5)	19 (9.5)	0.66
Laboratory tests, median (IQR)			
Creatinine level	89 (61–163)	86 (60–150)	0.386^∗^
Urea level	6.7 (3.9–12.8)	6.9 (4.2–13.6)	0.183^∗^
Kidney function (known abnormal) (mean ± SD)			
Creatinine level	404 ± 257	384 ± 267	0.682
Urea level	16.3 ± 8.3	19 ± 10	0.123
Kidney function (normal, abnormal) (mean ± SD)			
Creatinine level	83 ± 37.7	77.6 ± 29	0.271
Urea level	6.5 ± 5.2	6.7 ± 5.4	0.661
Kidney function status			
Normal	120 (60)	124 (62)	**< 0.001**
Abnormal	24 (12)	15 (7.5)	
Known abnormal	55 (27.5)	59 (29.5)	
Not done	1 (0.5)	2 (1)	
ICU length of stay (median, IQR)	24 patients 4 (1–12)	45 patients 8 (3.5–23.5)	**0.013**
Hospital length of stay (median, IQR)	5 (3–10)	8 (4–14)	**0.001**

*Note:* Statistically significant *p*-values < 0.05 are shown in bold.

^∗^Mann–Whitney *U* test.

^∗∗^Fisher exact test.

**Table 3:** National early warning scores [17] comparisons at key stages.

NEWS2 scale	First admission *n* (%)	First discharge *n* (%)	*p* value	First discharge *n* (%)	Second admission *n* (%)	*p* value
Low (0–2)	23 (11.5)	34 (17)	**< 0.001**	34 (17)	16 (8)	**< 0.001**
Moderate (3–5)	31 (15.5)	75 (37.5)		75 (37.5)	35 (17.5)	
High (6-7)	23 (11.5)	52 (26)		52 (26)	21 (11.5)	
Critical (≥ 8)	123 (61.5)	39 (19.5)		39 (19.5)	128 (64)	

*Note:* The significance of differences between groups was analyzed using the chi-square test. Statistically significant *p*-values < 0.05 are shown in bold.

## Data Availability

The data supporting this study's findings are available from the corresponding author [Salim Baharoon] upon reasonable request.
